# Role of Doppler Evaluation in Assessing the Maturation of the Arteriovenous Fistula for Hemodialysis: An Observational Study

**DOI:** 10.7759/cureus.55527

**Published:** 2024-03-04

**Authors:** HS Suraj, Sakalecha Anil Kumar, N Rachegowda, Govindaraju Tirupathi Rajeswari, L Yashas Ullas, RB Revanth

**Affiliations:** 1 Radiodiagnosis, Sri Devaraj Urs Academy of Higher Education and Research, Kolar, IND

**Keywords:** ultrasonography, doppler, arteriovenous fistulae, kidney diseases, dialysis

## Abstract

Introduction

Arteriovenous fistulas (AVFs) are the preferred type of vascular access for hemodialysis due to their lower risk of complications. This study aimed to determine the role of Doppler evaluation in assessing AVF.

Materials and methods

We conducted an 18-month prospective observational study of 33 hemodialysis patients who underwent a procedure for the creation of AVF at the Department of Radio-Diagnosis at Sri Devaraj Urs Academy of Higher Education and Research. Patients were evaluated using color Doppler ultrasound. Participants underwent a careful history and clinical examination to diagnose the disease. All relevant parameters were documented in a structured study proforma. AVF maturation was assessed postoperatively at four weeks using Doppler ultrasound color flow evaluation by looking for vascular components (flow volume, vein, and arterial diameter). Data were analyzed using CoGuide V 1.0.3 Statistical Software (CoGuide, Bangalore, India).

Results

A total of 33 patients, with a mean age of 54.6 ± 7.8 years, were evaluated. Of the 33 participants, 24 (72.7%) were male, and nine (27.3%) were female. The majority (47%, n=16) of participants had diabetes mellitus, eight (24%) had hypertension, and 10 (29%) had both diabetes mellitus and hypertension. A brachiocephalic fistula was created in 45.5% of participants, and 33.33% had radiocephalic anastomoses. Five participants were diagnosed with AVF complications: two had a pseudoaneurysm, and three had a cephalic vein thrombus. Clinical and demographic characteristics (age, vascular components, and complications) were not significantly related to AVF maturation.

Conclusions

Doppler ultrasound plays an important role in selecting vessels for AVF preoperatively and assessing AVF maturation postoperatively, thus reducing the primary fistula failure rate. The findings suggest that Doppler evaluation can be a reliable tool for assessing AVF maturation and predicting surgical success, which could help healthcare providers make informed decisions about the best course of treatment for their patients. Continued research is warranted in this area to further understand the role of Doppler ultrasound in evaluating AVF surgery.

## Introduction

Chronic kidney disease is India’s ninth-leading cause of death, responsible for 2.9% of all deaths among 15- to 69-year-olds [1]. The age-adjusted incidence of end-stage kidney disease (ESKD) in India is 226 per million, and approximately 220,000 new ESKD patients are added to the pool annually [2]. In India, most of these patients with ESKD die due to a lack of treatment [3]. Hemodialysis (HD) is a widespread practice worldwide for renal replacement therapy, and almost 63% of patients with ESKD depend on HD for their survival [4]. Success depends on numerous factors that contribute to access to HD and the clinical consequences of fistula failure [5].

Central venous catheters, arteriovenous grafts (AVG), and autologous arteriovenous fistulas (AVF) are the three most common methods of vascular access for HD. The creation of AVF has been recommended since 1997 among HD patients. The National Kidney Foundation in 2003 established the Fistula First Breakthrough Initiative, recommending 50% fistula rates for the first placed access (incident) and 40% for patients with previous surgically created accesses (prevalent, which has now changed to 68%) [6]. The native AVF is the most effective vascular access (VA) to ensure long-term survival and quality of life in HD patients and is recommended as the first choice for its extended survival and low infection rates [7]. Various methods of screening for AVF have been proposed, including physical examination, measurement of access blood flow, access recirculation, direct or derived static dialysis pressure, and color Doppler ultrasound (CDUS) [8].

Nephrologists are using CDUS as a direct tool for functional vascular assessment. It is a cheap and noninvasive technique that allows a detailed morphological and functional VA assessment to study superficial structures without contrast agents. With a decreased probability of primary failure of freshly produced AVF, more alternatives to performing native distal AVF, and higher efficacy in managing the prevailing dysfunctional AVF, a multidisciplinary approach in the consultation of vascular access with routine CDUS can have significant benefits for the patient. Furthermore, it can reduce health costs in fistulograms and phlebography by minimizing and optimizing complementary examinations. Additional research is needed to understand the true impact of multidisciplinary teams on the construction and function of AVF, patient quality of life, and healthcare costs. Therefore, this study aimed to determine the role of CDUS in assessing the maturation of AVF.

## Materials and methods

Study design

We conducted an 18-month descriptive observational study in the Department of Radio Diagnosis at Sri Devaraj Urs Academy of Higher Education and Research. The primary outcome was AVF maturation (success and failure rate), and AVF complications were secondary outcome variables. The study included hemodialysis patients referred after four weeks of AVF construction to the Department of Radio-Diagnosis at R.L. Jalapa Hospital and Research Center attached to Sri Devraj Urs Medical College, Tamaka, Kolar. Thirty-three eligible subjects were recruited into the study consecutively by convenience sampling until the sample size was reached. The sample size was estimated using the maturation rate of AVFs detected by CDUS using the method reported by Cases et al. [9] with the following formula:

$Z1-&alpha;/22 p(1-p)/d2a$

Where Z1-α/2 = 1.96 at 5% error alpha.

We considered p-values below 0.05 significant; therefore, 1.96 is used in the formula.

p = Expected proportion in the population based on previous or pilot studies.

d = Absolute error or precision (decided by the researcher).

p = 0.75 1 - p = 0.25 d = 15%

Using the above values at a 95% confidence level, a sample size of at least 33 subjects was determined.

The study was conducted following approval from the institutional research and ethics committees (Approval No. SDUMC/KLR/EC/135/2019-20). Study participants were informed of the protocol and the requirement of regular follow-up for completion of the study, and they provided informed consent to participate. The confidentiality of the study participants was maintained.

Inclusion and exclusion criteria

The study included patients with insufficient/no AVF flow during the dialysis session, patients with difficulty in cannulation, upper limb edema, persistent discomfort and/or pain in the fistula arm, and patients with a subcutaneous venous collateral formation in the upper limb or thoracic wall. The study excluded patients with upper limb arterial and venous occlusive disorders, patients with deformed or scarred upper limbs, and patients whose vascular anatomy did not permit the construction of a native AVF (according to preoperative CDUS).

Data collection

The participants underwent a careful history assessment and clinical examination to determine the diagnosis of the disease and the requirement for creating native AVFs. All relevant parameters were documented in a structured study proforma. Postoperative follow-up was performed after four weeks of AVF construction using a CDUS scanner (PHILIPS EPIQ 5G, Philips Ultrasound, Bothell, WA) by a dedicated, qualified radiologist (three years of experience).

For both the first and subsequent access, we followed the National Kidney Foundation Kidney Disease Outcomes Quality Initiative guidelines, which provide recommendations for the preparation of the largest number of native AVFs, as well as supervision and monitoring programs (clinical and instrumental) for the identification of potentially treatable causes of VA failure to act quickly and prevent thrombosis [10].

AVF maturation

The maturation period is the time between the creation of AVF/AVG and the first cannulation, and different countries follow different periods. The median time for the first successful AVF cannulation was 10 days in Japan, 46 days in Europe, and 82 days in the United States [11]. Clinical maturation of AVF was defined as unassisted when not preceded by a percutaneous or surgical intervention to promote maturation and otherwise as assisted. Overall maturation was defined as clinical maturation with or without prior intervention [12]. AVF maturation was defined as the clinical use of the AVF with two needles for 75% of dialysis sessions over a continuous four-week period, including either a mean dialysis machine blood pump speed of >300 ml/min over four consecutive sessions or a measured Kt/V>1.4 or a urea reduction ratio (URR) >70% [13].

Doppler assessment procedure

The preferred site of access placement was determined by a Duplex Doppler ultrasound examination. Vessel caliber, wall morphology, peak systolic velocity (PSV) of the arteries, and patency of the vessels were assessed. A tourniquet was placed around the arm, and the superficial venous system (namely the cephalic, basilic, and median cubital veins) was assessed first in the transverse plane, followed by the longitudinal planes. The distal nondominant limb was first preferred, followed by the proximal nondominant and dominant limbs. Subsequently, the radial and brachial arteries’ arterial diameter and flow velocities were assessed. Postoperative follow-up was performed after the fourth week for all patients, with an emphasis on assessing ultrasound parameters of the venous component. The sites of AVF and arterial and venous components were evaluated with an ultrasound grayscale and CDUS examination. We documented the diameter, PSV, and flow volume (FV) of the venous component; the diameter and PSV of the arterial component; and the width, depth, and PSV in AVF [14].

Statistical methods

The descriptive analysis is presented as the mean and standard deviation (SD) for quantitative variables such as age. Categorical variables such as gender, clinical history, type of AVF, and AVF complications were presented as frequency and proportion. Categorical results were compared between study groups using the chi-square test. We considered p < 0.05 to be statistically significant. Data were analyzed using CoGuide V 1.0.3 Statistical Software (CoGuide, Bangalore, India) [15].

## Results

A total of 33 patients were included in the study. The mean age was 54.6 ± 7.8 years (range, 41 to 69 years). Most participants (42.42%, n=14) were between 50 and 59 years old. Twenty-four (72.7%) were male, and nine (27.3%) were female. Most participants (47%, n=16) had diabetes mellitus, eight (24%) had hypertension, and 10 (29%) had diabetes mellitus plus hypertension. Regarding AVF type, the most common was brachiocephalic anastomoses in 15 participants (45.4%), followed by radiocephalic anastomoses in 11 participants (33.3%). The mean diameter of the venous component was 4.9 mm (range, 4.55 to 5.6 mm), PSV was 111 cm/s (range, 91 to 145 cm/s), and FV was 466 mL/min (range, 364 to 1,024 mL/min). Five participants were diagnosed with AVF complications; two had a pseudoaneurysm and three had a cephalic vein thrombus. Successful AVF was reported in 10 participants (30.3%; Table 1). 

**Table 1:** Patient population analysis (N=33)

Parameter	Result
Mean age ± SD (years)	54.6 ± 7.8 (range, 41 to 69)
40 to 49, n (%)	9 (27.27%)
50 to 59, n (%)	14 (42.42%)
60 to 69, n (%)	10 (30.30%)
Sex	
Male, n (%)	24 (72.7%)
Female, n (%)	9 (27.3%)
Clinical history	
Diabetes mellitus, n (%)	16 (47%)
Hypertension, n (%)	8 (24%)
Diabetes mellitus and hypertension, n (%)	10 (29%)
Type of arteriovenous fistula	
Brachial-median cubital, n (%)	7 (21.2%)
Radiocephalic, n (%)	11 (33.3%)
Brachiocephalic, n (%)	15 (45.4%)
Mean Diameter - Venous Component (mm, n=33)	4.9 (4.55 to 5.6)
PSV - Venous Component (cm/sec, n=30)	111 (91 to 145)
FV - Venous Component (ml/min, n=30)	466 (364 to 1024)
Diameter - Arterial Component (mm, n=33)	5.4 (4.8 to 5.75)
PSV - Arterial Component (cm/sec, n=33)	286 (246.5 to 436)
Width at AVF (mm, n=33)	6.1 (5.5 to 6.5)
Depth of AVF (mm, n=33)	6.7 (6.05 to 7.45)
PSV At AVF (cm/sec, n=33)	375 (320 to 589)
Complication	
CV thrombus, n (%)	3 (60%)
Pseudoaneurysm, n (%)	2 (40%)
Status of AVF	
Failure to mature, n (%)	23 (69.7%)
Successful, n (%)	10 (30.3%)

Age was not significantly associated with AVF type (p=0.793). Of the seven participants with brachial-median cubital AVF, four reported failures to mature, and three reported successful AVF. Of the 15 participants with brachiocephalic AVF, seven reported a failure to mature, and six reported successful AVF. Of the 11 participants with brachiocephalic AVF, seven reported a failure to mature (Table 2).

**Table 2:** Comparison of baseline parameters across AVF types (N=33) *No statistical test was applied due to 0 subjects in the cells AVF: arteriovenous fistula; CV: cephalic vein

Parameter	Type of arteriovenous fistula			P-value
	Brachial- Median Cubital (N=7)	Brachiocephalic (N=15)	Radiocephalic (N=11)	
Age Group				
40 to 49 years, n (%)	2 (28.57%)	4 (26.67%)	3 (27.27%)	0.793
50 to 59 years, n (%)	3 (42.86%)	5 (33.33%)	6 (54.55%)	
60 to 69 years, n (%)	2 (28.57%)	6 (40%)	2 (18.18%)	
Status of AVF				
CV Thrombus, n (%)	1 (14.29%)	1 (6.67%)	1 (9.09%)	*
Failure to mature, n (%)	4 (57.14%)	7 (46.67%)	7 (63.64%)	
Pseudoaneurysm, n (%)	0 (0%)	1 (6.67%)	1 (9.09%)	
Successful, n (%)	2 (28.57%)	6 (40%)	2 (18.18%)	

AVF type was not significantly associated with postprocedure vascular diameters or flow rate. The mean postprocedure FV was 471 mL/min (range: 347.25 to 1,135.75 mL/min) in the brachial median cubical fistula, 441 mL/min (range: 362.25 to 689.25 mL/min) in the brachiocephalic fistula, and 475.5 mL/min (range: 367 to 1,740.5 mL/min) in the radiocephalic fistula (Table 3). 

**Table 3:** Comparison of ultrasound assessed parameters across AVF type (N=33) PSV: Peak systolic velocity; FV: Flow volume; AVF: Arteriovenous fistula

Parameter	Type of arteriovenous fistula			Kruskal Wallis Test (P-value)
	Brachial-Median (Range) Cubital (N=7)	Brachiocephalic-Median (Range) (N=15)	Radiocephalic Median (Range) (N=11)	
Diameter - Venous Component (N=33, mm)	5.4 (4.9, 5.5)	5.2 (4.7, 5.7)	4.7 (4.3, 5.1)	0.054
PSV - Venous Component (N=30, cm/sec)	108.5 (83, 146.75)	114 (94.75, 146.75)	114 (89, 144.75)	0.895
FV – flow volume Venous Component (N=30, mL/min)	471 (347.25, 1135.75)	441 (362.25, 689.25)	475.5 (367, 1740.5)	0.587
Diameter - Arterial Component (N=33, mm)	5.2 (4.4, 5.6)	5.6 (5.1, 5.8)	5.2 (4.8, 5.8)	0.385
PSV – Arterial Component (N=33, cm/sec)	267 (264, 423)	287 (246, 424)	286 (241, 457)	0.978
Width at AVF (N=33, mm)	6 (5.4, 6.3)	6.1 (5.9, 6.6)	5.9 (5.5, 6.6)	0.477
Depth of AVF (N=33, (mm)	7.5 (6.4, 8.5)	6.8 (5.8, 8.1)	6.5 (6, 6.9)	0.158
PSV At AVF (N=33, cm/sec)	347 (314, 601)	375 (322, 645)	399 (318, 584)	0.788

**Figure 1:**
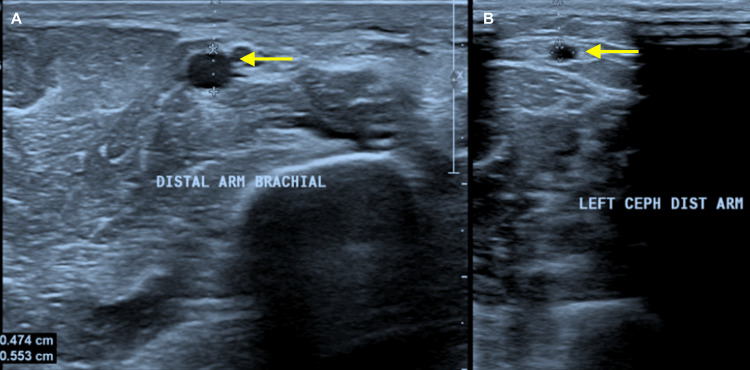
Ultrasound grayscale images showing preoperative arterial and venous mapping of the brachial artery (1A) and cephalic vein (1B) using a linear probe (3-12 MHz)

**Figure 2:**
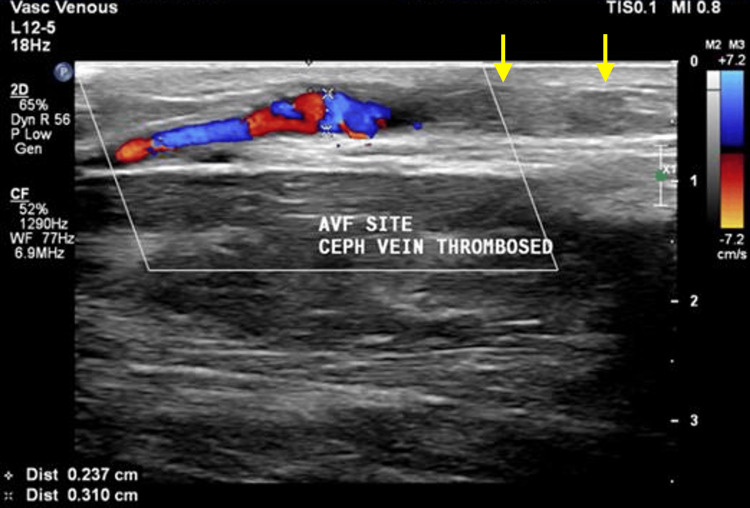
Color Doppler ultrasound image showing long segment venous thrombosis of the cephalic vein close to the site of AVF using a linear probe (3-12 MHz) AVF: Arteriovenous fistula

**Figure 3:**
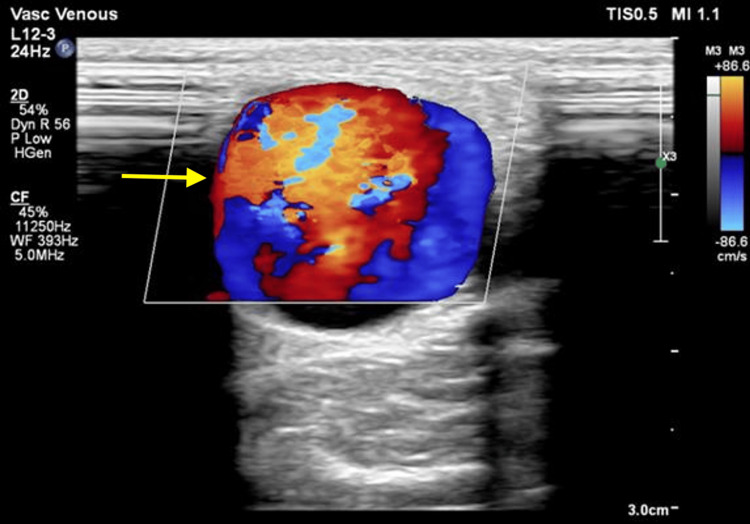
Color Doppler ultrasound showing pseudoaneurysm formation with a classic “Yin and Yang” pattern using a linear probe (3-12 MHz)

**Figure 4:**
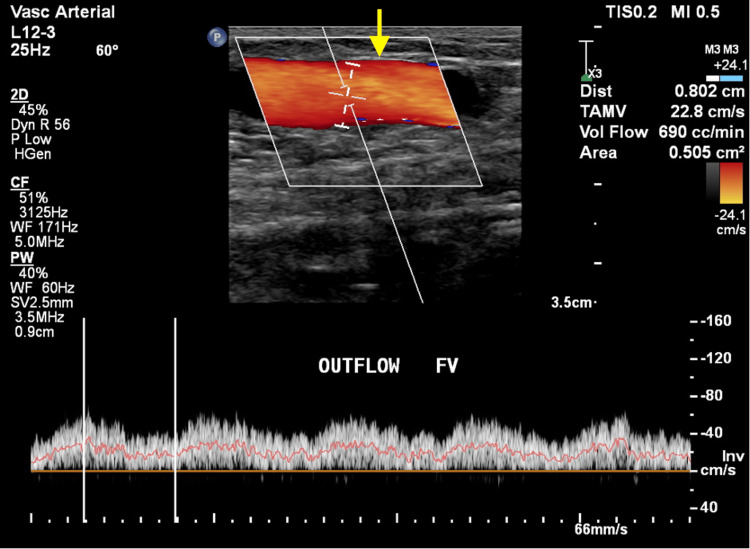
Color Doppler ultrasound image demonstrating flow volume (mL/min) of the outflow component using a linear probe (3-12MHz)

Figures 1A, 1B show grayscale ultrasound images of preoperative arterial and venous mapping of the brachial artery (1A) and cephalic vein (1B) using a linear probe (3 to 12 MHz). Figure 2 shows a CDUS of a long-segment venous thrombosis of the cephalic vein close to the AVF site using a linear probe (3 to 12 MHz). Figure 3 shows a CDUS of a pseudoaneurysm formation with a classic “yin and yang” pattern using a linear probe (3 to 12 MHz). Figure 4 shows a CDUS of the outflow component’s FV (mL/min) using a linear probe (3 to 12 MHz).

## Discussion

Within three months of surgery, nearly 30% of fistulas fail due to technical errors that cause thrombosis in the first 24 hours of surgery, and some failures occur due to insufficient blood flow [16]. Although the cause of most early failures is unclear, the quality of the vessels is considered to play an important role. Other causes can be small radial and cephalic arteries and veins that are atherosclerotic, stenosed, or partially thrombosed [17]. For adequate dialysis, only 60% of autogenous fistulas mature sufficiently. However, even after successful maturation, most cases require reintervention due to repeated trauma from percutaneous access and the vexing problem of flow stenosis [18]. CDUS helps identify preoperative and postoperative vascular mapping for AVF creation, assess the prime time for a puncture, detect complications of AVF, such as an abscess, aneurysm, steal syndrome, and hematoma, and identify appropriate therapeutic procedures [19]. Mudoni et al. found that ultrasound can reduce the number of subsequent invasive angiographic procedures and requires interdisciplinary cooperation in AVF monitoring; they suggested including monitoring as part of an integrated vascular access management program [20]. Prasad et al. found that the early detection of AVF/AVG with low probabilities of maturation helps with earlier intervention by enhancing or even replacing it with a new VA [21]. The duration of exposure to central venous catheters and associated morbidity can be reduced by early detection of such cases.

This is the first study to determine the role of Doppler evaluation in assessing the maturation of AVF in a tertiary care center. The need for AVF maintenance has naturally led to this initial operation. In this study, the overall success rate of AVF was only 10 (30.30%), with a nearly 70% failure rate. The complication rate was much lower, and cephalic vein thrombus was the most common complication. The brachiocephalic vein was the most common site, and 40% of participants had successful placement.

Clinical and demographic characteristics (e.g., age, vascular components, and complications) were not significantly related to fistula maturation. Dageforde et al. found no significant association with other demographic and clinical characteristics such as age, sex, body mass index, peripheral vascular disease, cardiovascular disease, or race [22]. Our overall AVF success rate was only 30.30%, with a near 70% failure rate. This contrasted with Zhu et al.’s study report that 14.4% (19/132) of AVFs failed to mature [23]. In Zhu et al.’s study, after ultrasound examination and recording of the parameters of the cephalic vein via the skin, the AVFs received successful cannulation. The reason for success can be the physical examination of AVF maturation before the first cannulation. It includes raising the arm to see whether the AVF collapses; if it does not collapse, it indicates either an outflow stenosis or a high-flow AVF. On palpation, a pulsatile AVF suggests a stenosis in the outflow segment. On auscultation, the presence of stenosis results in a high-pitched predominantly systolic bruit distal to the stenosis, followed by a return of normal bruit proximal to the stenosis. Normal mature AVF has a continuous bruit that can be heard in systole and diastole. Clinically, some AVFs are mature and suitable for cannulation. Therefore, the objective evaluation of the maturity of AVF with Doppler parameters is of great clinical importance.

In our study, vascular components such as the diameter, width, and depth of the vein and artery of the AVF were considered in assessing AVF maturation. Robbin et al. found the diameter and volume flow at two weeks, accurately predicting six weeks with de novo autogenous fistulas [24]. We found an increase in FV and draining vein diameter. Similarly, Fonseca et al. found an increase in both FV and draining vein diameter [25], thus confirming the existence of a maturation process for better vascular access.

Data in the literature on CDUS FV assessment indicate that a well-functioning AVF will be characterized by a flow rate of 700 to 1,300 mL/min. Our study found a mean FV at four weeks of 475.5 mL/min, ranging from 367 to 1,740.5. Flow <500 mL/min and <300 mL/min are considered predictive of access dysfunction and imminent thrombosis, respectively [26]. Our FV and drainage vein diameter of 5 mm were adequate for HD sessions.

We also performed Doppler preoperative venous mapping to avoid complications. El Khoury et al. found no clinically helpful predictor of fistula maturation and reported that successful primary maturation does not depend on routine preoperative venous mapping [27]. Han A et al. found no significant differences in the maturation failure rate between Doppler ultrasound surveillance and physical examination by an experienced vascular surgeon [28].

Our study had several important limitations. We only assessed primary failures. Secondary failures were not evaluated because they require long-term follow-up; this was a short-term follow-up study. We also did not account for other factors that determine the success of AVF maturation, such as the surgeon’s experience level. Our small sample size and single-center location limit the applicability of our results to other populations. An intraoperative CDUS assessment of arteriovenous anastomoses would be more helpful in predicting maturity; however, this was not available in our facility. More multicentric longitudinal studies with large samples are recommended to validate the present study’s findings.

## Conclusions

The current study focused on the early postoperative evaluation of AVF maturation using CDUS to reduce the maturation failure rate and the timely management of any detected lesions. Current data do not support any conclusion on whether CDUS assessment is beneficial, even in the early postoperative period. A careful physical examination by an expert or a combination of CDUS and physical examination may be as effective and more economical in the early identification of AVF with abnormalities during maturation. CDUS plays an essential role in selecting vessels for AVF preoperatively, which reduces the rate of primary fistula failure, as well as in assessing the postoperative maturation of AVF. Peripheral vascular access for HD remains an area of medicine with high unmet clinical needs. This technique would help healthcare professionals bring relevant information to the management of AVF.
